# A non-destructive evaluation strategy for surface weathering degree of limestone cultural relics in Zhejiang, China

**DOI:** 10.1371/journal.pone.0332696

**Published:** 2025-09-17

**Authors:** Yongguo Chen, Zixuan Chen, Zhenlei Wu, Liang Ye, Zhiwei Pan, Mengsen Fang, Jichuan Zheng

**Affiliations:** 1 School of Civil Engineering and Architecture, Zhejiang University of Science and Technology, Hangzhou, China; 2 Zhejiang- Singapore Joint Laboratory for Urban Renewal and Future City, Hangzhou, China; 3 Longyou Museum, Longyou, China; China University of Mining and Technology, CHINA

## Abstract

In order to fully assess the protection of Limestone cultural relics from all forms of deterioration in Zhejiang Province, this study explored the relationship between the strength, longitudinal wave velocity and water absorption of limestone surface through indoor and field tests, and obtained a set of scientific evaluation methods for evaluating the surface weathering degree of limestone cultural relics. The results show: the surface layer strength of limestone and water absorption, longitudinal wave velocity and water absorption have obvious laws, good fitting effect, all have strong negative correlation; the humidity environment has a greater effect on the water absorption of limestone surface layer, the lower the humidity of the test environment, the more obvious the pattern between the strength of limestone surface layer, longitudinal wave velocity and water absorption. The study of water absorption on rock surfaces concluded that water absorption is closely related to the degree of weathering, which plays an important role in the further conservation of limestone cultural relics.

## Introduction

In the ever-expanding field of heritage conservation, the issue of surface deterioration of cultural relics is becoming increasingly prominent. The majority of stone cultural relics in open environments are subjected to various natural weathering processes and acid rain erosion, including temperature and humidity fluctuations, atmospheric exposure, microbial activity, and solar radiation, which lead to cracks of varying degrees on the surface of the relics. Dorina et al. confirmed through long-term monitoring that the microclimate (temperature 20 ± 2°C, RH 60–70%) within traditional stone buildings is a hotbed for fungal growth (>800 CFU/m^3^), which is highly similar to the environment in which limestone artifacts are found in Zhejiang [1]. Currently, there exists a multitude of methods for assessing the degree of weathering in stone cultural relics, which qualitatively evaluate factors such as the color, integrity, mineral composition, chemical composition, and hydrophysical properties of the stone. However, most of these methods provide only qualitative analysis rather than quantitative data, failing to accurately capture the mechanical properties of the stone’s surface layer. This limitation significantly restricts their utility in informing subsequent protective measures for the cultural relics [2].

In the past decades, many researchers have tried to obtain the degree of weathering of rock surfaces using simple and reliable predictive models. For example, a regression model for predicting the degree of rock weathering was developed by combining terahertz spectroscopy and ultrasonic velocimetry [3]. Rocky monuments are facing the threat of weathering, and it is very important to accurately define the weathering characteristics for protection. The integrated weathering classification was proposed to identify the degree of weathering by combining non-destructive methods such as visual analysis and P-wave velocity data. This method eliminates the classification boundary through the fuzzy inference system and realizes the collective evaluation [4]. Using hyperspectral imaging technology and deep learning network CNN, 19,456 granite sample data were used to achieve a reliable assessment of the degree of rock weathering, with an average accuracy of more than 94%. This method is practical and accurate, without relying on the experience or prejudice of professionals [5]. Developing a weathering index that reveals the correlation between mechanical and engineering geological behaviour to enable more accurate predictive models for geological hazards [6]. The development of an intrinsic damage model that better reflects the deformation and damage process of rocks under dry and wet loading conditions under repeated dry and wet cycling [7]. A good correlation between the physico-mechanical properties and dry, saturated tuff was indicated by one-way regression analysis [8,9]. Scholars have conducted in-depth explorations into the complex properties of weathered rocks. Okewale et al. through the comparison of volcanic rock samples, revealed the influence of the geological features of the parent rock on the weathering process, despite the fact that the structural features of weathered rocks are independent of those of the parent rock [10]. Yasir et al. studied the mineralogy, structure, and weathering grades of intrusive igneous rocks in northern Pakistan, along with their impact on physical and strength properties. They discovered that different rock types exhibit distinct physical and strength characteristics [11]. On the other hand. D’Addario et al. discovered a correlation between the degree of weathering and engineering geological properties of trachytic volcanic rocks in the Monte Amiata region of central Italy. Furthermore, they proposed a quantitative method for assessing the degree of weathering through thermal analysis [12]. These studies contribute significantly to our understanding and prediction of the engineering behavior of weathered rocks.

There are also scholars who have studied the internal structure and properties of rocks and their surroundings to determine the degree of weathering. For example, the structural safety of infrastructures built on carbonate rocks was investigated under saturated conditions and structural safety assessment was carried out using three-dimensional finite element modelling [13]. The profile longitudinal wave velocity distribution of bridge stone masonry was examined by ultrasonic CT technique to determine the degree of weathering of slag based on the ratio of the longitudinal wave velocity of slag to that of fresh rock [14,15]. To study the weathering characteristics of acidic volcanic rocks and their influence on rock engineering properties, and to establish a weathering grading system for acidic volcanic materials [16,17]. Determining the water absorption characteristics of natural building stones of different composition, structure and texture, it was found that the porosity of highly absorbent stones is also an accelerating factor in the drying process [18] Mechanical properties of fly ash and its control mechanism, significant differences in disintegration amount and disintegration rate of soil samples treated with different curing agents were obtained [19] Infrared thermal imaging, an indirect moisture assessment technique, has been used to provide important information for the study of moisture in porous materials by complementary investigations of the microstructure and isothermal behaviour of stone [20,21]. The damage induced during the excavation of a test tunnel in granite was inferred from the distribution of microcracks consistent with microseismic activity during the excavation by means of impulse source transducers at multiple points on the borehole and tunnel surface [22].

The primary objectives of this work are to: develop a field-applicable NDE protocol using water absorption as a key proxy for weathering severity;quantify the influence of environmental humidity (20°C/60–70% RH) on measurement reliability; establish quantitative thresholds for classifying weathering degrees (slight/moderate/strong) in Zhejiang limestone heritage. The novelty of this study lies in the introduction of a novel non-destructive evaluation approach that offers a more comprehensive assessment of the weathering degree of limestone cultural relics. Using a fully non-destructive, convenient, and environmentally adaptable experimental apparatus, we explore the relationship between water absorption and surface layer strength, as well as the variation between water absorption and longitudinal wave velocity, in limestone specimens with varying degrees of weathering. By detecting surface layer strength, longitudinal wave velocity, and water absorption, the proposed evaluation method allows for a more convenient and non-destructive determination of the weathering degree of stone cultural relics. Furthermore, this approach provides valuable insights for the preliminary preservation of cultural heritage.

## Materials and methods

**Fig 1:**
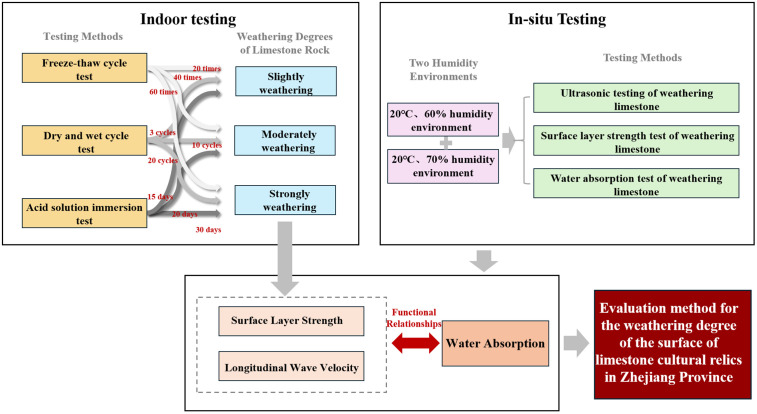
Research workflow integrating laboratory simulation and field validation.

The research followed a dual-track approach integrating laboratory simulations and field validation, as visualized in **Fig 1**.

### Materials

**Fig 2:**
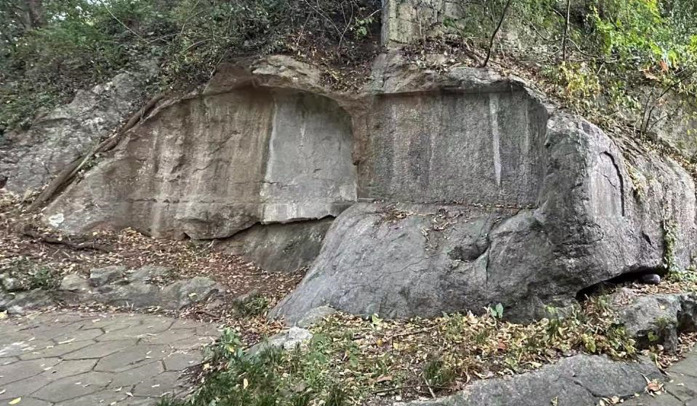
Geographical location of the study region.

**Fig 2** shows the statue of Baifoyan in Wushan Scenic Area, Ziyang Street, Shangcheng District, Hangzhou City, Zhejiang Province, China. Geographical coordinates:30°14’11.6“ north latitude, 120°9’43.1” east longitude, altitude: 60.00 meters. Ziyang Mountain in Wushan Scenic Area belongs to karst low-hill landform. The stratum is Carboniferous Chuanshan Formation limestone, gray-gray-black limestone, locally containing purplish red coarse-grained limestone, and the rock is sedimentary rock.

In this experiment, an evaluation method based on limestone mass weathering is proposed through indoor tests and in-situ testing experiments. Through the investigation of the disease of the surface layer of limestone cultural relics in Zhejiang area, it is found that the freeze-thaw cycle, the dry-wet cycle and the erosion of acidic solution are the main factors leading to the weathering of limestone, and based on the results of the investigation of the disease, we design a simulated weathering experiment which is in line with the real environment of limestone in Zhejiang area, so as to improve the reliability of the experimental data [23,24]. However, the number of rock samples in the indoor test is small, and the fitting function is not persuasive enough. To address this problem, the limestone specimens were tested in situ, and enough testing data were obtained through acoustic test, rebound test, and water absorption test to establish a real and reliable fitting function [25].

**Fig 3:**
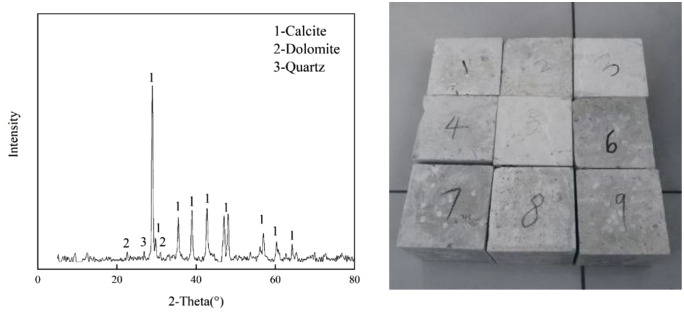
Xray diffraction spectra and samples.

**Fig 3** fresh rock samples were ground into powder and placed on the sample stage of the X-ray diffractometer, and the absorption peaks of Calcite and a small amount of Dolomite in the samples were obtained by detection, which showed that the samples were mainly composed of CaCO_3_ and SiO_2_, as well as other small amounts of impurities.The samples measure 100 mm*100 mm*100 mm. The water content, water absorption, rebound value, compressive strength, surface layer strength and longitudinal wave velocity of the specimens under natural conditions were tested by compressive strength tester, JC-50 moisture meter, RSM-SY7 ultrasonic detector, HT-225 A rebound meter and other instruments in turn. The average value of the test data of 9 rock samples was taken as the standard value of fresh limestone.

To investigate the influence of humidity on water absorption, two different humidity environments were selected to test the water absorption of limestone, specifically 20°C/60% humidity and 20°C/70% humidity. Based on these data, the selection criteria are as follows: According to the China Statistical Yearbook published by the National Bureau of Statistics, the average temperature in Hangzhou in 2020 was 18.3°C, with an average humidity of 73%, and the humidity range was between 60% and 85%. In 2021, the average temperature in Hangzhou was 18.8°C, with an average humidity of 71%. Both annual average temperatures are close to 20°C, thus the test temperature was determined to be 20°C. Months with an average relative humidity ranging from 60% to 75% account for approximately 80% of the total number of months, therefore, 60% and 70% were chosen as the humidity environments for testing. Finally, the temperature and humidity conditions for testing were determined to be 20°C with 60% humidity and 20°C with 70% humidity, as these two temperature and humidity patterns are applicable for a relatively long period of time.

### Indoor testing methods

Three types of simulated weathering experiments were carried out on limestone rock samples through dry and wet cycles, freeze and thaw cycles, and acid and alkaline salt solution immersion, and three different weathering degrees of limestone rock samples, namely, slightly weathering, moderately weathering, and strongly weathering, were finally obtained [26].

### Freeze-thaw cycle test

Rock freeze-thaw cycle test involves key indicators such as freeze-thaw cycle temperature, duration, and number of cycles [27,28]. Prior studies have established a relationship between the weathering degree of limestone and the number of freeze-thaw cycles, with 40 cycles reducing the strength of grey rock to 80% of its fresh state, indicating slight weathering according to the *Code for Investigation of the Protection Engineering of Stone Monument* (GB 50021−2001(2009)_0625) [29–31]. The simulated weathering test involves freeze-thaw cycles set at 20, 40, and 60 times for slightly, moderately, and strongly weathered stones. The test involves grouping stones by weathering degree, immersing limestone samples in water for 48 hours, boiling to saturate, wiping with gauze, freeze-thaw cycles were conducted using a programmable environmental chamber (Model GDJW-100C, Dongguan Bell Experiment Equipment Co., China) with temperature range −40°C to +150°C (±0.5°C accuracy). Specimens were placed in stainless steel mesh racks (304 SS, mesh size 5 × 5 mm) to ensure uniform air/water contact. Thawing utilized a thermostatic water bath (Model HH-4, Jintan Yitong Instrument Co., China) equipped with titanium alloy heating elements and PID temperature control (±0.3°C accuracy).

### Dry and wet cycle test

Wet/dry cycle is considered as a wet/dry cycle by immersing the rock specimen in water for 24 h and then drying it in a drying oven at 105°C for 24 h [32–34]. For the simulated weathering test, the number of dry-wet cycles is specifically set to simulate different degrees of weathering: 3 cycles for slightly weathering, 10 cycles for moderately weathering, and 20 cycles for strongly weathering. The limestone rock samples are divided into three groups: slightly weathering, moderately weathering, and strongly weathering, according to the simulated weathering targets. Each group undergoes the specified number of dry-wet cycles as described above.

### Acid solution immersion test

Limestone belongs to carbonate rock, which will react with acidic solutions such as nitric acid and sulfuric acid. According to the 2021 Hangzhou Ecological Environment Bulletin, the acid rain in Hangzhou is mainly sulfuric acid rain. Limestone immersed in acidic solution with pH = 1.5, the trend of its wave velocity is divided into three stages, showing a first decline, followed by a short rise and then a continuous decline trend [35]. After soaking limestone for 5 days, porosity changes significantly. By day 10, compactness increases due to internal mineral particles reacting with water, creating larger volumes that fill pores. After 10 days, porosity continues to decline until day 15, when limestone reaches slight weathering. By day 20, moderate weathering occurs. Acid immersion employed corrosion-resistant PTFE containers (200 × 200 × 150 mm, Chemours Co., USA) to prevent secondary contamination. pH = 1.5 sulfuric acid solution was prepared with 18.2 MΩ·cm deionized water (Milli-Q Advantage A10, Merck KGaA, Germany) and analytical-grade H₂SO₄ (99.999%, Sigma-Aldrich). pH stability was monitored by a digital meter (Model PHB-4, Shanghai INESA Co., China) with glass electrode (accuracy ±0.01 pH).

### In-situ testing methods

The acoustic properties, surface layer strength and water absorption of the rock samples were tested in different humidity environments. In order to study the effect of environment humidity on water absorption, two different humidity environments were chosen to test the water absorption of limestone, namely 20°C 60% humidity environment and 20°C 70% humidity environment. The surface layer strength, longitudinal wave velocity, and water absorption of the limestone surrounding the Baifo Rock Statue were measured across various humidity levels. Subsequently, the collected data were analyzed using Origin software to establish the functional relationships between limestone surface layer strength, longitudinal wave velocity, and water absorption under different humidity conditions.

Permission Statement: Fieldwork and field testing were conducted at the White Buddha Temple stone statue site (Hangzhou Wushan Scenic Spot) under official permission from the Huagang Management Office of Hangzhou West Lake Scenic Area. The Huagang Management Office of Hangzhou West Lake Scenic Area, as the statutory authority for the site, allowed access to the site and oversaw the non-destructive testing procedures to ensure compliance with heritage conservation protocols. The non-invasive measurements (acoustic, rebound, and surface moisture tests) conducted in this study did not require additional environmental or geologic permits.

### Performance test of limestone with different weathering degree

#### Ultrasonic testing of weathering limestone.

Rock longitudinal wave velocity detection adopts RSM--SY7 ultrasonic detector of Zhongyan Technology. The two probes and the coupling agent are closely attached to the surface of the rock to be tested, and the coupling agent is butter. When the instrument is started, the time scale value corresponding to the first jumping point of the waveform curve displayed on the fluorescent screen can be taken as the time of arrival of the longitudinal wave, and the ratio of the distance to the time can be used to Eq. (1)


$$V_{p}=\frac{s}{t_{p}-t_{s}}$$ (1)

The formula:

*V*_*P*_-Longitudinal wave velocity, in meters per second (m/s);

*S*-Acoustic wave propagation distance in meters (m);

*t*_*p*_-Longitudinal wave arrival time in microseconds (μs);

*t*_*s*_-Zero error of the instrument system, in microseconds (μs).

After measuring the longitudinal wave velocity of the rock samples, the degree of weathering of each rock sample was judged according to the method of determining the degree of rock weathering based on ultrasonic longitudinal wave velocity of the Specification for Investigation of Stone Cultural Relics Conservation Projects (WW/T 0063–2015), i.e., **Table 1** and Eq. (2)

**Table 1:** Rock weathering coefficient Fs weathering degree grading.

Degree of weathering	Rock weathering coefficient
Unweathering	*F*_*S*_ < 0.1
Slightly weathering	0.1 ≤ *F*_*S*_ < 0.25
Moderately weathering	0.25 ≤ *F*_*S*_ < 0.5
Strongly weathering	0.5 ≤ *F*_*S*_


$$Fs=\frac{V_{p0}-V_{p}}{V_{p}}$$ (2)

The formula:

*Fs* -Rock weathering coefficient;

*V*_*p0*_ – Fresh rock longitudinal wave velocity (m/s);

*V*_*P*_ – Weathering rock longitudinal wave velocity (m/s).

#### Surface layer strength test of weathering limestone.

Surface strength was measured by HT-225A digital rebound hammer (Beijing Huatian Testing Instrument Co., China) conforming to IS0 8045:2020. Key parameters: impact energy: 2.207 J (±0.5%), hammer mass: 0.55 kg with tungsten carbide impact tip (hardness 90 HRA), displacement sensor: Laser-encoded optical encoder with 0.1 R resolution, calibration was performed daily using certified anvil blocks (Rockwell hardness HRC 60 ± 2). The axis of the rebound instrument is always perpendicular to the surface of the test piece, the rebound instrument downward striking, the striking rod is divided into four rotations, each rotation of 90°, striking 3–5 times, take the last of which three consecutive readings of the rebound value of the average of the stable. The product of the rebound value and the rock’s density exhibits a linear relationship with the rock’s compressive strength. Therefore, by measuring the rebound value and density, the compressive strength of the rock can be estimated. Through the rebound meter’s measurement of the rock surface’s rebound value, a linear relationship with the surface layer strength is observed, which aligns with the objective of this study to investigate the degree of weathering on the rock surface. This further confirms that the surface layer strength has a significant impact on the degree of weathering [29]. With reference to the *code for investigation of the protection engineering of stone monument* (GB 50021−2001(2009)_0625) based on the weathering of rock and fresh rock strength ratio of rock weathering grade, the ratio of 0.9-1.0 are divided into unweathering rocks, the ratio of 0.8-0.9 are classified as slightly weathering rocks, the ratio of 0.4-0.8 are classified as moderately weathering rocks, the ratio of less than 0.4 rocks are classified as strongly weathering rocks.

#### Water absorption test of weathering limestone.

In this paper, water absorption was quantified via JC-50 electromagnetic moisture meter (Shenzhen Jietong Technology Co., China) with these characteristics: Frequency: 1 GHz TDR (Time Domain Reflectometry), probe: 35 mm diameter flat coil sensor (stainless steel 316L), depth resolution: 5–50 mm adjustable, calibrated against gravimetric measurements (ASTM D2216-19), accuracy: ± 0.5% for 0–5% moisture range. Each measurement applied 10N contact pressure using integrated spring-loaded arm. [29]. The water absorption of rocks is affected by the humidity of the environment in which they are located. Therefore, when establishing a method for evaluating the weathering degree of limestone artefacts based on the water absorption property, it is necessary to consider the influence of different humidity environments on the water absorption property of the limestone surface layer [36,37].

## Results and discussion

### Fresh rock sample test results

Through the basic physical test of rock samples, the test results of fresh rock samples can be obtained, as shown in **Table 2**. The water content, water absorption, rebound value, compressive strength, surface layer strength and longitudinal wave velocity of the specimens under natural conditions were tested by compressive strength tester, JC-50 moisture meter, RSM-SY7 ultrasonic detector, HT-225 A rebound meter and other instruments in turn. The average value of the test data of 9 rock samples was taken as the standard value of fresh limestone.

**Table 2:** Properties of fresh limestone specimens.

Serial number	Weight G (g)	20°C/60% Humidity water absorption (%)	20°C/70% Humidity water absorption (%)	Rebound value (R)	Converted compressive strength value (MPa)	*V*_*p0*_(m/s)	Densityρ(g/cm^3^)
Test block 1	2466	0.11	0.07	56	137	4571	2.47
Test block 2	2468	0.16	0.15	56	137	4167	2.47
Test block 3	2511	0.13	0.11	57	144	4545	2.51
Test block 4	2503	0.09	0.09	56	137	4434	2.50
Test block 5	2479	0.06	0.06	57	144	4671	2.48
Test block 6	2457	0.13	0.14	57	144	4545	2.46
Test block 7	2491	0.15	0.11	56	137	4431	2.49
Test block 8	2471	0.13	0.15	56	137	4545	2.47
Test block 9	2468	0.09	0.09	56	137	4571	2.47
Average value	2579.3	5.54	0.12	56.30	139.30	4498	2.48

#### Indoor test results.

Ultrasonic test results of weathering limestone. Comparing the testing data of limestone rock samples from each simulated ageing group, the limestone rock samples from all three simulated ageing groups reached the set degree of weathering, and the purpose of the ageing experiment was achieved, as shown in **Table 3**.

**Table 3:** Application of ultrasonic testing for longitudinal wave velocity in simulated weathering rock samples.

Serial number	*V*_*P*_(m/s)	*V*_*p0*_ (m/s)	rock weathering coefficient *Fs*	Simulated weathering experiment group
1	4023	4571	0.12	Slightly weathering
2	3846	4167	0.17	
3	3703.5	4545	0.22	
4	3573.5	4434	0.26	Moderately weathering
5	3448.5	4671	0.30	
6	3333.5	4545	0.35	
7	2913	4431	0.54	Strongly weathering
8	2935	4545	0.53	
9	2879	4571	0.56	

Weathering limestone strength determination. Using the HT-225A rebound hammer, strength testing was conducted on weathering limestone. The *code for investigation of the protection engineering of stone monument* stipulates a method for converting the rebound value of rock masses into uniaxial compressive strength [29]. According to the rebound test data of the limestone rock samples, the limestone rock samples of each simulated aging group reached the corresponding range of strength ratios as shown in **Table 4**, and the purpose of the aging experiment was achieved.

**Table 4:** Strength test of weathering rock samples.

Serial number	Mean value of rebound (R)	Converted uniaxial compressive strength (MPa)	Converted uniaxial compressive strength before test (MPa)	Rock weathering coefficient *Fs*	Simulated weathering experiment group
1	52.7	117.6	137	0.84	Slightly weathering
2	53.1	119.7	137	0.86	
3	51.6	111.6	144	0.80	
4	47.3	90.5	137	0.65	Moderately weathering
5	48.6	96.4	144	0.69	
6	44.0	77.0	144	0.55	
7	37.8	59.3	137	0.42	Strongly weathering
8	35.1	52.3	137	0.37	
9	36.0	54.7	137	0.39	

Determination of water absorption of weathering limestone. The water absorption properties of rocks are inevitably influenced by the humidity of their environment. Therefore, when establishing an evaluation method for the weathering degree of limestone cultural relics based on water absorption characteristics, it is necessary to consider the influence of different humidity environments on the water absorption characteristics of limestone. The *code for investigation of the protection engineering of stone monument* points out that the humidity environment of stone cultural relics can be simulated through watering [29]. The test was performed twice, before and after sprinkling. The sprinkling method was spraying with a spray bottle, the amount of water was 200 ml each time, the spraying distance was 1m, and the spraying method was holding the spray bottle perpendicular to the surface of the rock, there was no more water traces on the surface of the rock in about 10 min after sprinkling, and then the second water content test was carried out at this time. Under the same temperature and humidity environment, the larger the increase in water content of limestone after sprinkling was, the looser the surface of the rock body, the higher the porosity between the rock particles, and the higher the degree of weathering of the rock. After 10 tests on the six surfaces of the weathering rock samples before and after moisturizing, the values were obtained by subtracting the average value of the surface water content before and after water sprinkling. It was regarded as the value of the water absorption of the rock in a certain period of time, and the average value of the 10 tests was taken as the value of the water absorption of the weathering rock samples as a whole, as shown in **Table 5**.

**Table 5:** Water absorption test of weathering rock samples.

Serial number	Water absorption at 20°C/60% humidity environment (%)	Water absorption at 20°C/70% humidity environment (%)	Water absorption at 20°C/60% humidity environment before test (%)	Water absorption at 20°C/70% humidity environment before test (%)	Degree of weathering of rock samples
1	0.59	0.25	0.11	0.07	Slightly weathering
2	0.82	0.51	0.16	0.15	
3	0.88	0.91	0.13	0.11	
4	0.63	0.7	0.09	0.09	Moderately weathering
5	1.1	0.85	0.06	0.06	
6	1.26	0.96	0.13	0.14	
7	1.61	1.58	0.15	0.11	Strongly weathering
8	2.03	1.89	0.13	0.15	
9	1.94	1.71	0.09	0.09	

#### In-situ test results.

Acoustic test results and analysis. Perform acoustic performance testing on Baifo Rock, and the results are shown in **Table 6**. The acoustic test results show that the first alcove of Baifo Rock has the highest degree of weathering, which belongs to strongly weathering limestone, and the weathering degree of the second alcove is lower than that of the first alcove and higher than that of the third alcove, which is between slightly weathering and moderately weathering, and is inclined to be moderately weathering. The third alcove has the lowest degree of weathering and is a slightly weathering limestone. In the acoustic test results of the first alcove, the measured longitudinal wave velocity are too low at some points due to the presence of deep weathering fissures, as shown in **Fig 2**.

**Table 6:** Longitudinal wave velocity test results.

Test area	Number of points	longitudinal wave velocity range (m/s)	Average value (m/s)	Degree of weathering
1	15	1855-2775	2583	Strongly weathering
2	10	2805-3199	3054	Moderately weathering
3	10	2704-3413	3237	Moderately weathering
4	20	3812-4588	3934	Slightly weathering
5	20	3215-3789	3437	Moderately weathering
6	15	3599-4267	3787	Slightly weathering

Surface rebound strength test results and analysis. The rebound test results for the six tested areas of the Baifo Rock are shown in **Table 7**. After converting the rebound values to compressive strength values, it shows that the weathering of the three alcoves is consistent with the acoustic test results.

**Table 7:** Surface rebound strength test results.

Test area	Check numbers	Rebound value (R)	Rock surface layer strength (MPa)	Average intensity (MPa)	Degree of weathering
1	15	30-41	33-67	51.2	Strongly weathering
2	10	38-42	61-70	67.2	Moderately weathering
3	10	39-48	63-95	82.7	Moderately weathering
4	20	51-58	109-153	131	Slightly weathering
5	20	49-54	77-126	99.3	Moderately weathering
6	15	47-56	88-137	117.6	Slightly weathering

Water absorption test results and analysis. The test results of the water content increase in the rock body of the Hundred Buddha Rock Statue shows that the higher the weathering degree of the area, the greater the water content increase value of the trend is. The water content increase value of the surface layer of the rock body and the longitudinal wave velocity and intensity correlate with the water content increase value and the longitudinal wave velocity. Intensity of the wave has an obvious negative correlation and the indoor simulation of the weathering test results are consistent with the results, as shown in **Table 8**.

**Table 8:** Water absorption test results.

Test area	Check numbers	Water absorption at 60% humidity (%)	Water absorption at 70% humidity (%)	Degree of weathering
1	15	1.93-4.22	1.84-3.51	Strongly weathering
2	10	1.72-2.17	1.78-1.91	Moderately weathering
3	10	0.99-1.80	1.48-1.6	Moderately weathering
4	20	0.19-0.55	0.12-0.52	Slightly weathering
5	30	0.78-1.23	0.65-1.37	Moderately weathering
6	15	0.43-0.93	0.31-0.7	Slightly weathering

### Analysis of indoor test results

#### Analysis of the results of the change rule of surface layer strength and water absorption.

**Fig 4:**
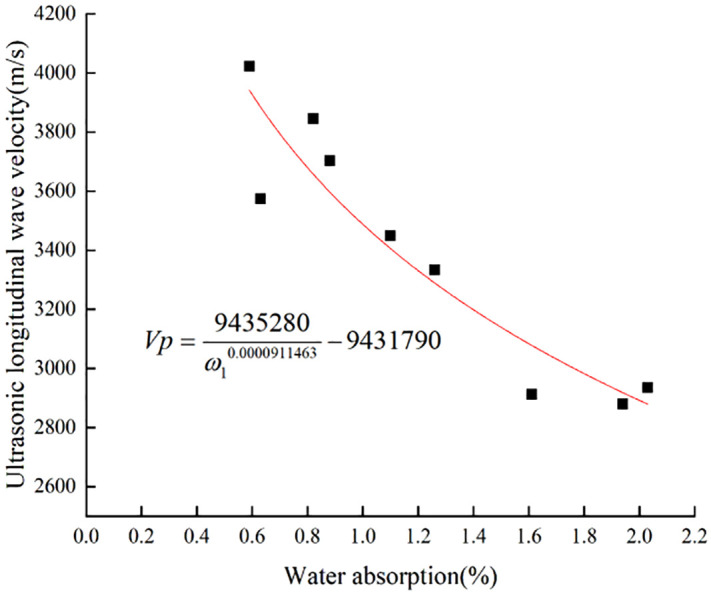
Longitudinal wave velocity-absorption variation rule of limestone at 60% ambient humidity.

**Fig 5:**
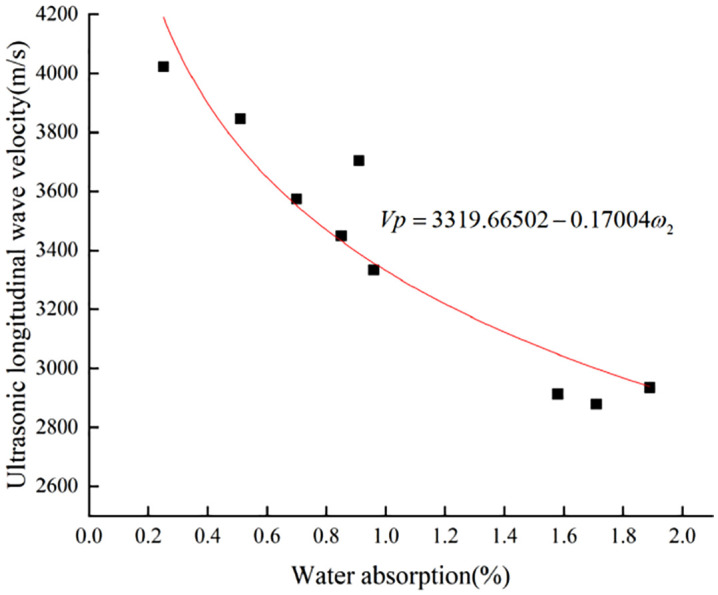
Longitudinal wave velocity-absorption variation rule of limestone at 70% ambient humidity.


The longitudinal wave velocity of limestone samples with different degrees of weathering shows an obvious negative correlation with water absorption. The effect of humidity on the water absorption of rocks can be seen from **Fig 4** and **Fig 5** the higher the ambient humidity, the lower the water absorption of rocks, and the lower the correlation with the longitudinal wave velocity. Different ambient humidity affects the accuracy of the evaluation method based on the water absorption [38]. The changing law of longitudinal wave velocity and water absorption under different humidity environments was fitted. According to **Fig 4** and **Fig 5**, the fitting function model Eq. (3) similar to the trend of the scatter plot of the detection data was selected. During the fitting process, it was found that the fitting function under 70% humidity environment could not converge, and the fitting function model Eq. (3), which is similar to Eq. (4), was selected. Finally, the fitting function Eqs. (5) and (6) were obtained. The coefficients of determination, R^2^ were 0.87 and 0.85, respectively. The coefficients of determination were between 0 and 1, and the closer they were to 1, the more accurate the fitting effect. It is generally recognized that there is a strong correlation between two variables with a coefficient of determination above 0.5. From the fitting results of the detected data, it is feasible to use the change rule of limestone water absorption and longitudinal wave velocity to judge the weathering degree of limestone. Park et al. [39] investigated the variation of water absorption and longitudinal wave velocity by increasing rock physical parameters with freeze-thaw cycles. By constantly freezing and thawing conditions, it was concluded that the relationship between water absorption and longitudinal wave velocity has a high correlation, providing meaningful linear fitting equations, and it was hypothesized that the degree of rock weathering can be detected by water absorption and longitudinal wave velocity, which is consistent with the study of rock weathering in the present paper, and it further confirms the significant effect of water absorption and longitudinal wave velocity on rock weathering.

$$y=\frac{a}{x^{b}}+c$$ (3)

$$y=ax^{b}$$ (4)

$$Vp=\frac{9435280}{{\omega_{1}}^{0.0000911463}}-9431790$$ (5)

$$Vp=3319.66502-0.17004\omega_{2}$$ (6)

The formulas:

*V*_*P*_-Ultrasonic longitudinal wave velocity of limestone (m/s);

*ω1, ω2-*Water absorption of limestone at 60% and 70% ambient humidity (%).

#### Analysis of the results of the change rule of longitudinal wave velocity and water absorption.

**Fig 6:**
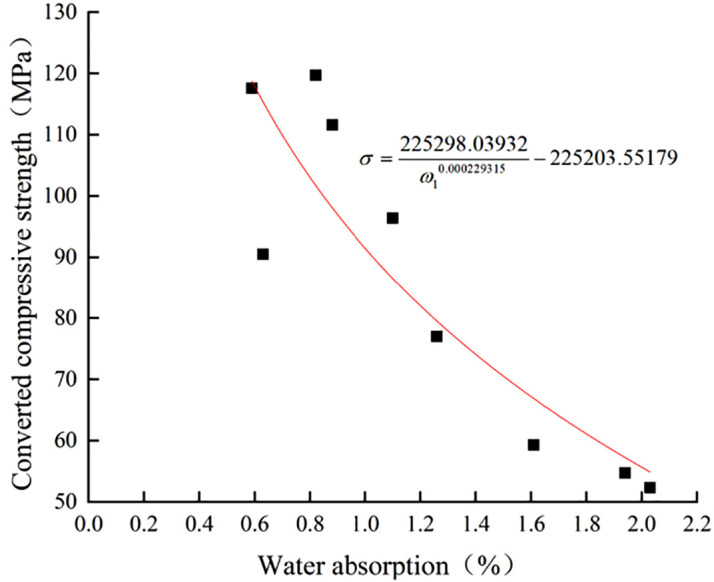
Surface layer strength-absorption pattern of limestone at 60% ambient humidity.

**Fig 7:**
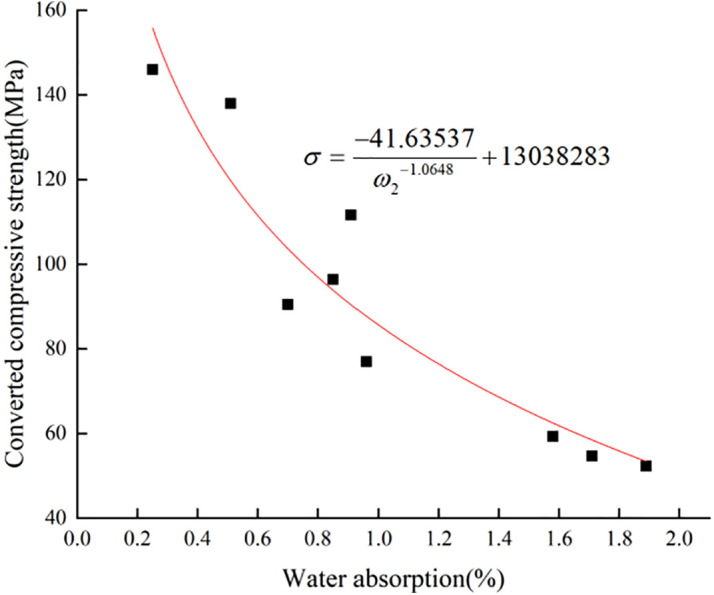
Surface layer strength-absorption patterns of limestone at 70% ambient humidity.


There is also an obvious negative correlation between the surface layer strength and water absorption of limestone samples with different degrees of weathering, and the test data are shown in **Fig 6** and **Fig 7**. The two patterns of change are fitted, and the fitted function model is the same as the wave velocity fitted model [40–41]. The Eq.s (7) and (8) were obtained under different environmental humidity, and the coefficients of determination R^2^ were 0.77 and 0.86, respectively.The surface layer strength of limestone rock samples has a strong correlation with the water absorption, and the surface layer strength of limestone samples exhibits a strong correlation with water absorption, and the patterns of change in these properties can reflect the degree of weathering of limestone. Meng et al. [42] studied the mechanism and potential control measures of safety hazards triggered by expanding rocks in coal mine engineering, and used a self-developed rock water absorption test system to carry out water absorption experiments at different temperatures to study the relationship between the water absorption rate and the compressive strength, and finally concluded that the compressive strength was negatively and linearly correlated with the water content. This is consistent with the law of rock weathering studied in this paper, and further confirms the significant influence of water absorption rate and longitudinal wave velocity on rock weathering.

$$\sigma =\frac{225298.03932}{{\omega_{1}}^{0.000229315}}-225203.55179$$ (7)

$$\sigma =\frac{-41.63537}{{\omega_{2}}^{-1.0648}}+13038283$$ (8)

The formulas:

$\omega_{1}$, $\omega_{2}$ -Water absorption of limestone at 60% and 70% ambient humidity (%);

σ -Converted compressive strength of limestone (MPa).

The mechanical properties and water absorption test data of limestone specimens with different degrees of weathering under different temperature and humidity environments are shown in **Fig 4** to **Fig 7** and **Table 9**. The uniaxial compressive strength, longitudinal wave velocity and water absorption of limestone with different degrees of weathering show obvious negative correlation, the higher the water absorption, the lower the uniaxial compressive strength and ultrasonic longitudinal wave velocity of limestone, and vice versa, the higher it is. The effect humidity of the environment on the water absorption performance of the rock is manifested in the fact that the higher the ambient humidity the worse the water absorption ability of the rock. The water absorption of limestone in 70% humidity environment is 25.7% lower than that in 60% humidity environment.

**Table 9:** Surface properties of limestone specimens after simulated weathering.

serial number	Average rebound value	Converted compressive strength (MPa)	longitudinal wave velocity (m/s)	Water absorption at 60% humidity (%)	Water absorption at 70% humidity (%)
1	57	146	4023	0.59	0.25
2	56	138	3846	0.82	0.51
3	52	112	3703	0.74	0.47
4	47	91	3504	0.88	0.91
5	49	96	3574	0.63	0.7
6	44	77	3448	0.82	0.61
7	38	59	2713	1.26	0.85
8	35	52	2735	1.1	0.96
9	36	55	2679	1.2	0.71

By fitting the detection data of surface layer strength and water absorption and longitudinal wave velocity and water absorption under different temperature and humidity environments, the coefficients of determination of the fitting functions are all greater than 0.8, indicating that the correlation between surface layer strength, longitudinal wave velocity and water absorption of limestone are extremely great. It is feasible to judge the weathering degree of limestone by water absorption as well as surface layer strength.

### Analysis of in-situ test results

#### Analysis of the results of the change rule of surface layer strength and water absorption.

**Fig 8:**
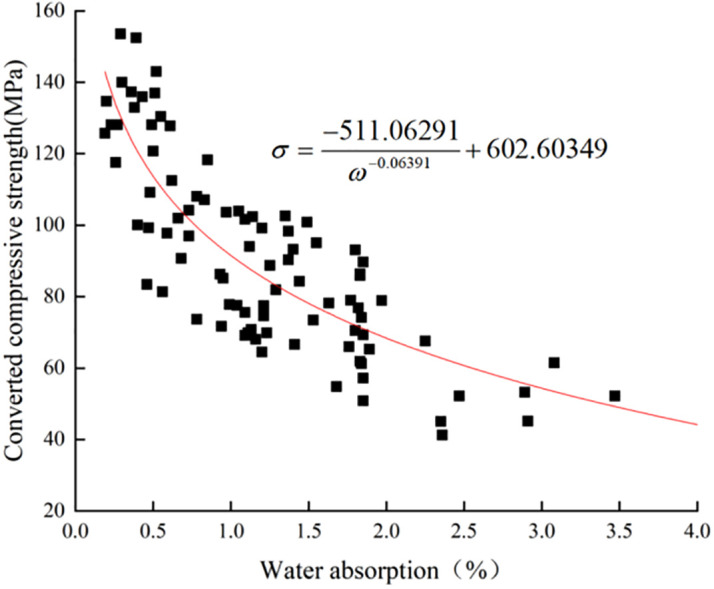
20°C/60% Limestone surface layer strength vs. water absorption fits.


The trend of the graph of strength versus water absorption of limestone surface layer in temperature 20°C/humidity 60% environment obtained by origin software is shown in **Fig 8**. it is similar to the image of function y = 1/x. The fitting function model is $y=\frac{A}{x^{B}}+C$ selected, the initial values of the fitting parameters A, B and C are taken as 1. Through the Levenber-Marquardt iterative algorithm, the nonlinear fitting function Eq. (9) is obtained. The coefficient of determination of the fitting function is R^2^ = 0.77, it can be used to measure the effect of the data fitting, and the closer the R^2^ is to 1, the better the effect of the fitting [29].

$$\sigma =\frac{-511.06291}{\omega^{-0.06391}}+602.60349$$ (9)

The formula:

ω -Water absorption (%)

σ -Converted compressive strength (MPa).

**Fig 9:**
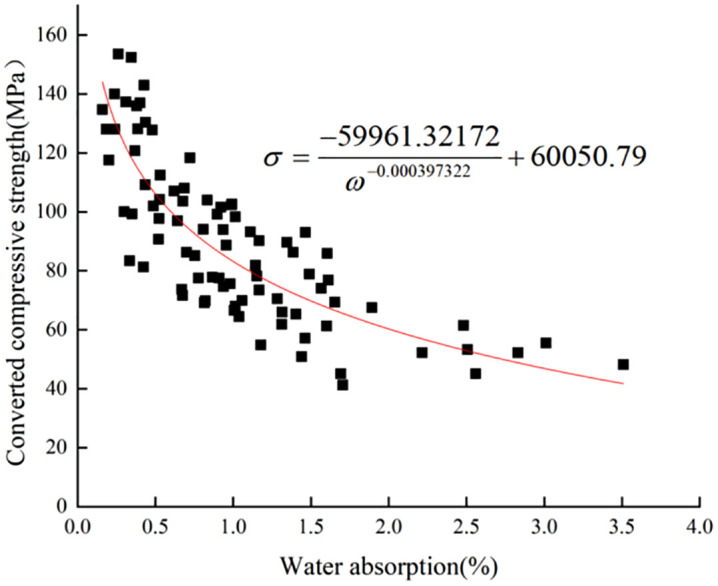
20°C/70% Limestone surface layer strength vs. water absorption fits.


The trend of the strength-absorption relationship graph of limestone surface layer under 20°C/70% humidity environment was obtained by origin software as **Fig 9**. The strength-absorption data were fitted, and the selection of fitting parameters and iterative algorithm was the same as in the previous section. The fitting converged after 528 iterations, and a nonlinear fitting function was obtained as Eq. (10), with the coefficient of determination of the fitting function R^2^ = 0.71.

$$\sigma =\frac{-59961.32172}{\omega^{-0.000397322}}+60050.79$$ (10)

The formula:

ω -Water absorption (%);

σ -Converted compressive strength (MPa).

The fit of the test data in 60% humidity environment is better than that in 70% humidity environment, and the humidity environment has some influence on the water absorption characteristics of limestone. This phenomenon aligns with paleoclimatic studies indicating that low-humidity regimes enhance the sensitivity of carbonate rock properties to hydric interactions [43]. It is hypothesized that it is due to the high water absorption capacity of limestone when the ambient humidity is low. The higher the ambient humidity, the closer the value of water content in the natural state of the rock is to the upper limit of saturation, which leads to a limitation of the water absorption capacity within the same period of time. Therefore, the lower the humidity at the time of testing, the more accurate is the pattern obtained.

#### Analysis of the results of the change rule of longitudinal wave velocity and water absorption.

**Fig 10:**
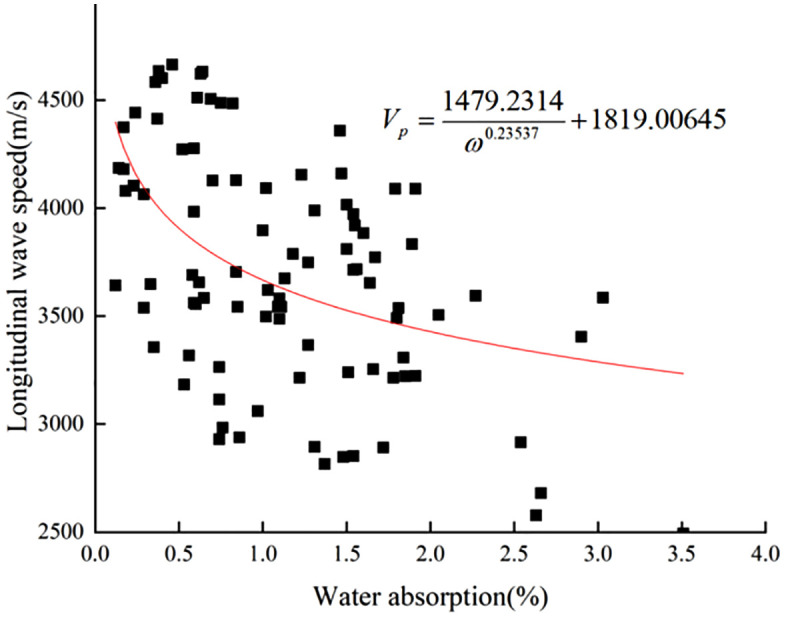
20°C/60% Humidity ambient limestone longitudinal wave velocity vs water absorption fitting plot.


The ultrasonic detection points are the same as the surface intensity detection points. Similar to the image of the function y = 1/x, so is the fitting function model. Fitting parameters, and iterative algorithm were selected as in the previous section. The fitting converged after 532 iterations to obtain the nonlinear fitting function Eq. (11) and **Fig 10** with a coefficient of determination of R^2^ = 0.34.The correlation between the longitudinal wave velocity and the water absorption of the in-situ testing of limestone was lower than that of the indoor test data, which is presumed to be due to the natural environment of the specimens surface layer complex disease and the effect of weathering fissures resulted.

$$V_{p}=\frac{1479.2314}{\omega^{0.23537}}+1819.00645$$ (11)

The formula:

ω -Water absorption (%);

*V*_*P*_-Rock longitudinal wave velocity (m/s).

**Fig 11:**
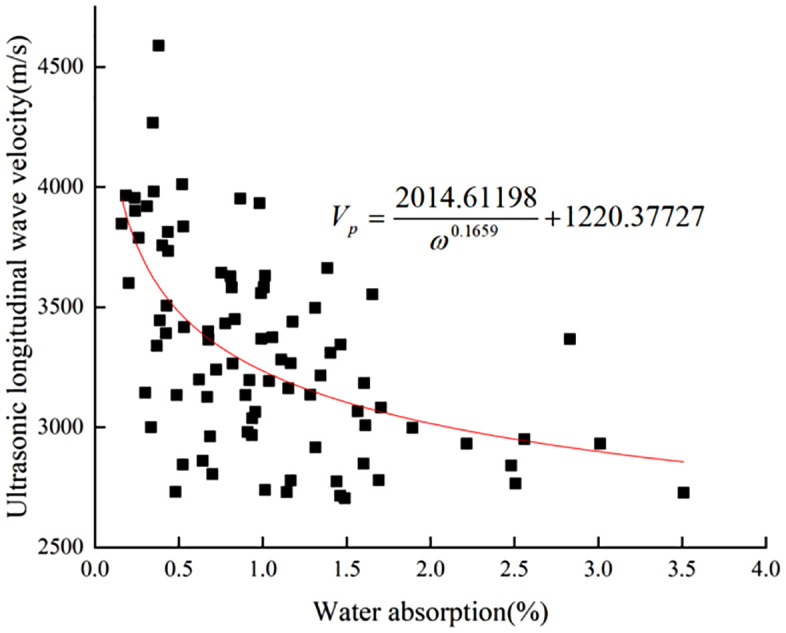
20°C/70% Humidity environment limestone longitudinal wave velocity vs water absorption fitting plot.


According to the detection data of ultrasonic longitudinal wave velocity and water absorption of limestone under 20°C/70% humidity environment is similar to the image of the function y = 1/x, so is the fitting function model. Fitting parameters, and iterative algorithm were selected as in the previous paper, and the fitting converged after 26 iterations to obtain the nonlinear fitting function Eq. (12) and the fitting **Fig 11**, with the coefficient of determination R^2^ = 0.34.

$$V_{p}=\frac{2014.61198}{\omega^{0.1659}}+1220.37727$$ (12)

The formula:

ω -Water absorption rate of surface layer (%);

*V*_*P*_-Rock longitudinal wave velocity (m/s).

In-situ tests revealed that the regularity between ultrasonic longitudinal wave velocity and water absorption in limestone in natural environment is poor and not as good as that obtained from laboratory tests. It is presumed to be due to the influence of some weathering fissures or complex diseases on the rock surface. The humidity environment has an effect on the water absorption of limestone, which mainly shows that the higher the ambient humidity, the worse the water absorption ability of limestone. The surface water absorption of 70% humidity environment is 15.7% lower than that of 60% humidity environment, and the trend is similar to the indoor test, but the decrease is smaller.

The ultrasonic longitudinal wave velocity and water absorption detection results of limestone in two different humidity environments showed similar trends of the surface layer strength and water absorption detection results. The surface layer strength and longitudinal wave velocity of limestone are negatively correlated with water absorption, and the fit of strength-absorption data is better than that of longitudinal wave velocity-absorption, which is presumed to be due to the inaccuracy of longitudinal wave velocity measurements because of the influence of some weathering fissures or complex deterioration on the rock surface. The humidity environment has a greater effect on the water absorption of limestone. The effect of humidity environment on the water absorption of the rock shows that the higher the ambient humidity, the worse the water absorption ability of limestone, the water absorption of 70% humidity environment compared with 60% humidity environment decreased by 10.7%, the trend is similar to the indoor test, but the rate of decrease is smaller.

### Method for evaluating limestone weathering degree based on water absorption

Existing codes employ three methods to evaluate rock weathering degree, two relying on longitudinal wave velocity and one on uniaxial compressive strength. In situ tests conducted on limestone’s surface layer strength, longitudinal wave velocity, and water absorption revealed that the correlation between limestone’s longitudinal wave velocity and water absorption was weak, while the correlation between surface layer strength and water absorption ranged from moderate to strong, as demonstrated in **Table 10**. Consequently, a weathering degree evaluation method was established based on the relationship between limestone’s surface layer strength and water absorption.

**Table 10:** Table of fitted functions.

Variables	Fit function	Coefficient of determination
Surface layer strength and water absorption (60% humidity environment)	$\begin{array}{c} \sigma =\frac{-511.06291}{\omega^{-0.06391}}+602.60349 \end{array}$	0.77
Surface layer strength vs. water absorption (70% humidity environment)	$\begin{array}{c} \sigma =\frac{-59961.32172}{\omega^{-0.000397322}}+60050.79 \end{array}$	0.71
Longitudinal wave velocity vs. water absorption (60% humidity environment)	$\begin{array}{c} V_{p}=\frac{1479.2314}{\omega^{0.23537}}+1819.00645 \end{array}$	0.34
Longitudinal wave velocity vs. water absorption (70% humidity environment)	$\begin{array}{c} V_{p}=\frac{2014.61198}{\omega^{0.1659}}+1220.37727 \end{array}$	0.34

The *Code for Investigation of the Protection Engineering of Stone Monument* (GB 50021−2001(2009)_0625) specifies a range of ratios between the strengths of rocks with varying degrees of weathering and the strength of fresh rocks. By combining the method outlined in the code with the fitting Eq. (9) and Eq. (10), a limestone cultural relics weathering degree evaluation method based on surface layer strength and water absorption can be derived for two different humidity environments.

(1) 20 °C 60% humidity environment: unweathered limestone specimens have a water absorption range of 0% to 0.25%; slightly weathered limestone specimens have a water absorption range of 0.25% to 0.41%; moderately weathered limestone specimens have a water absorption range of 0.41% to 2.56%; strongly weathered limestone specimens have a water absorption greater than 2.56%.
(2) 20 °C 70% humidity environment: unweathered limestone specimens have an absorption range of 0% to 0.15%; slightly weathered limestone specimens have a water absorption range of 0.15% to 0.28%; moderately weathered limestone specimens have a water absorption range of 0.28% to 3.44%; strongly weathered limestone specimens have a water absorption greater than 3.44%.

## Conclusions

This study investigates the current state of limestone cultural relics in Zhejiang Province. Currently, there is a lack of targeted methods for assessing the weathering degree of limestone cultural relics, and effective quantitative evaluation techniques have yet to be established. Therefore, this study simulates the weathering process of limestone through indoor experiments, examines the data on limestone surface layer strength, longitudinal wave velocity, and water absorption, determines the fitting effect of the strength-water absorption relationship, and establishes an evaluation method for the surface weathering degree of limestone cultural relics in combination with existing standards. The following results are obtained:

(1) An evaluation method for the surface weathering degree of limestone cultural relics based primarily on water absorption was established through indoor experiments and existing standards. By studying the data on limestone surface layer strength, longitudinal wave velocity, and water absorption, it is proven that the method of based primarily on water absorption is feasible. Experimental results indicate that there is a strong negative correlation between the surface layer strength, water absorption, and longitudinal wave velocity of limestone under different humidity conditions.
(2) The humidity environment significantly impacts the water absorption of limestone. The lower the humidity of the test environment, the more apparent the relationship between the surface layer strength, longitudinal wave velocity, and water absorption of limestone. Higher humidity levels result in poorer water absorption capacity of limestone, limiting the accuracy of the water absorption-based weathering degree assessment method. This is because environmental humidity affects the natural water content of rocks, further influencing their water absorption capacity.
(3) In-situ test reveal that there are differences in the correlation between ultrasonic longitudinal wave velocity and water absorption compared to indoor tests. This indicates that in practical applications, it is necessary to comprehensively consider multiple factors to more accurately assess the weathering degree of limestone cultural relics.

There are many other limitations to this study, such as the humidity thresholds (60% and 70% RH at 20°C) were only validated in the subtropical climate of Zhejiang. For arid regions (e.g., Northwest China) or tropical coastal regions (e.g., Hainan), the need to recalibrate the absorption criteria due to different evaporation dynamics, etc. needs to be considered.
